# Long-term dietary nitrate supplementation slows the progression of established atherosclerosis in ApoE^−/−^ mice fed a high fat diet

**DOI:** 10.1007/s00394-023-03127-7

**Published:** 2023-02-28

**Authors:** Yang Liu, Kevin D. Croft, Trevor A. Mori, Tracey A. Gaspari, Barbara K. Kemp-Harper, Natalie C. Ward

**Affiliations:** 1 School of Biomedical Sciences, University of Western Australia, Perth, WA Australia; 2 Medical School, University of Western Australia, Perth, WA Australia; 3 Department of Pharmacology, Monash Biomedicine Discovery Institute, Monash University, Clayton, VIC Australia; 4 Dobney Hypertension Centre, Medical School, University of Western Australia, G.P.O Box X2213, Perth, WA 6847 Australia

**Keywords:** Nitrate, Cardiovascular disease, Atherosclerosis, ApoE^−/−^ mice

## Abstract

**Background and aims:**

Atherosclerosis is associated with a reduction in the bioavailability and/or bioactivity of endogenous nitric oxide (NO). Dietary nitrate has been proposed as an alternate source when endogenous NO production is reduced. Our previous study demonstrated a protective effect of dietary nitrate on the development of atherosclerosis in the apoE^−/−^ mouse model. However most patients do not present clinically until well after the disease is established. The aims of this study were to determine whether chronic dietary nitrate supplementation can prevent or reverse the progression of atherosclerosis after disease is already established, as well as to explore the underlying mechanism of these cardiovascular protective effects.

**Methods:**

60 apoE^−/−^ mice were given a high fat diet (HFD) for 12 weeks to allow for the development of atherosclerosis. The mice were then randomized to (i) control group (HFD + 1 mmol/kg/day NaCl), (ii) moderate-dose group (HFD +1 mmol/kg/day NaNO3), or (iii) high-dose group (HFD + 10 mmol/kg/day NaNO3) (20/group) for a further 12 weeks. A group of apoE^−/−^ mice (n = 20) consumed a normal laboratory chow diet for 24 weeks and were included as a reference group.

**Results:**

Long-term supplementation with high dose nitrate resulted in ~ 50% reduction in plaque lesion area. Collagen expression and smooth muscle accumulation were increased, and lipid deposition and macrophage accumulation were reduced within atherosclerotic plaques of mice supplemented with high dose nitrate. These changes were associated with an increase in nitrite reductase as well as activation of the endogenous eNOS-NO pathway.

**Conclusion:**

Long-term high dose nitrate significantly attenuated the progression of established atherosclerosis in the apoE^−/−^ mice fed a HFD. This appears to be mediated in part through a XOR-dependent reduction of nitrate to NO, as well as enhanced eNOS activation via increased Akt and eNOS phosphorylation.

**Supplementary Information:**

The online version contains supplementary material available at 10.1007/s00394-023-03127-7.

## Introduction

Cardiovascular disease (CVD) is a major contributor to global morbidity and mortality [1]. Endothelial dysfunction, caused by impaired bioavailability and/or bioactivity of the vasoprotective molecule nitric oxide (NO) is the initial step in the development of atherosclerosis [2]. Endothelial dysfunction is characterized by reduced NO, chronic inflammation of the arterial wall, plaque formation and progressive occlusion [3]. NO is produced by three different isoforms of the enzyme NO synthase (NOS) via the classical _L_-arginine-NOS pathway, with endothelial NOS (eNOS) the major isoform responsible for maintaining vascular tone [4]. Animal and human studies of CVD have demonstrated reductions in eNOS-derived NO bioavailability [5], which is a key pathogenic mechanism involved in the formation and progression of atherosclerotic plaque [6]. In addition to endogenous NO generation through the conversion of _L_-arginine to citrulline, NO is also produced via the NOS-independent nitrate-nitrite-NO pathway [7]. Unlike the _L_-arginine-NOS pathway, the nitrate-nitrite-NO pathway is oxygen independent and regarded as an alternate source of NO during ischemia/hypoxia [8, 9].

Epidemiological studies have demonstrated that increased intake of green leafy vegetables, which are rich in nitrate, can improve endothelial function and may provide a way to modulate CVD development [10–12]. Dietary supplementation with nitrate has also been shown to have beneficial effects on vascular function in humans and in animal models of hypertension, diabetes and atherosclerosis [6, 13–17]. Our previous study showed that long term dietary nitrate supplementation had significant beneficial effects on acetycholine-mediated vascular function in apoE^−/−^ mice fed a HFD. This was accompanied by a reduction in plaque size and an increase in plaque stability, demonstrating a protective effect of dietary nitrate in the development of atherosclerosis [18]. However, the majority of patients only become aware of their atherosclerosis when it presents as a cardiac event. Plaque rupture is the main cause of such an event and can result in a myocardial infarction or stroke. Consequently, treatments that can prevent or reverse the progression of atherosclerosis after disease is already established, will have greater translational impact in human studies. The present study aimed to establish if long-term dietary nitrate supplementation could prevent or attenuate the progression of atherosclerosis after disease establishment, whether the effects were dose-dependent and the mechanisms behind any potential beneficial effects.

## Materials and methods

### Animal study

Eighty male apoE^−/−^ mice (6–8 weeks of age) were acclimatized for 1 week before 60 mice were switched to a HFD (50% carbohydrate, 21% fat supplemented with 0.15% cholesterol via addition of clarified butter (ghee), and 23% protein, Glenn Forrest Stockfeeds, WA). The remaining 20 mice were continued on a normal laboratory diet (NLD, 7% simple sugars, 4.5% fat with 0.02% cholesterol, 50% polysaccharide, 15% protein, Specialty Feeds, meat free rat and mouse diet) for the duration of the study as a reference group. Following 12 weeks on a HFD to allow for the development of atherosclerosis [18], mice were randomly divided into three groups (n = 20/group): (i) control group (HFD diet +1 mmol/kg/day NaCl, [HFD+NaCl]), (ii) moderate-dose group (HFD diet +1 mmol/kg/day NaNO_3_, [HFD+MDN]), and (iii) high-dose group (HFD diet +10 mmol/kg/day NaNO_3_, [HFD+HDN]). Treatments were given in the drinking water and mice were maintained on the supplemented diets for an additional 12 weeks. Body weight and food intake were measured weekly. The project was approved by the Royal Perth Hospital Animal Ethics Committee (R539/18-21) and Perkins Animal Ethics Committee (AE154). All experiments were conducted in accordance with the NHMRC guidelines for the care and use of laboratory animals in Australia. The study is reported in accordance with ARRIVE guidelines.

### Serum lipid profile and biochemistry

At the end of the treatment period, fasted (5 h) mice were anesthetized by inhalation of isoflurane (2% in 100% oxygen). Blood samples were collected by cardiac puncture and stored at 4 °C overnight to allow the blood to clot. Serum was separated via centrifugation (4000 rpm for 10 min at 4 °C) and stored at − 80 °C for later analysis. The serum levels of triglycerides (TG), total cholesterol (TC), high-density lipoprotein cholesterol (HDL-C), and low-density lipoprotein cholesterol (LDL-C) were analysed using standard colorimetric methods by PathWest Laboratories (Perth, WA). Serum endothelin (Enzo Lifesciences, NY, USA, #ADI-900-020A) and leptin (R&D systems, MN, USA, SM0B00B) were measured using ELISA kits, with all analysis conducted according to manufacturer instructions. Serum cGMP concentrations were measured using an ELIZA kit (Enzo Lifesciences, NY, USA, #ADI-900-164).

### Serum NO_x_ measurements

Serum nitrate and nitrite concentration was assessed using a gas chromatography mass spectrometry (GCMS) method as previously described [19].

### Plaque characterization

The entire aorta was excised, and the surrounding fat removed. Atherosclerotic lesion area in the cross-sections of brachiocephalic arteries (BCA) were paraffin wax-embedded, sectioned and stained with H&E (CellCentral, UWA). The aortic root and aortic ring close to the thoracic-abdominal region were isolated and placed in a cryomould filled with optimum cutting temperature (OCT) compound, frozen on dry ice and stored at − 80 °C for subsequent analysis. Further sectioning and staining with P-IκBα (1:200 dilution, Cell Signalling, #28595) as a marker of nuclear factor kappa B (NFκB) activation, the pan-macrophage marker CD68 (1:200 dilution, Thermofisher; BS-0649R) and alpha smooth muscle actin (1:500 dilution, αSMA; Abcam, ab5694) as a marker of vascular smooth muscle cells. Picrosirius red (PSR) staining was performed to determine the amount of collagen and Oil Red O (ORO) staining was utilized to determine the presence of lipid-laden plaques in the aortic sections. These techniques were performed as previously described [20, 21].

### Tissue protein expression

Protein expression of *p*-eNOS^ser1177^ (#9571, 1:500), AMPK (#2603, 1:500), *p*-AMPK^thr172^ (#2535, 1:500), Akt (#4691, 1:500), *p*-Akt^ser473^ (#4060, 1:500) (Cell Signaling Technology, MA, USA), eNOS (BD Biosciences, CA, USA, #610298, 1:500) and HO-1(Enzo Lifesciences, #ADI-SPA-895-F,1:500) were determined via western blot in isolated aortic samples as previously described [22]. Expression of xanthine oxidoreductase (XOR) (Abcam, VIC, AU, Ab231316, 1:500) was determined in both aorta and liver via western blot. To minimize interference between antibodies, all membranes were cut according to molecular weight size prior to incubation with primary antibody.

### Statistical analysis

Data processing was performed with Graphpad Prism (version 9). All results are shown as mean ± SEM. All analysis was conducted using one-way ANOVA with Duncan’s post-hoc comparisons. Repeated measures analysis was performed on data collected over the 24 week study period for weight gain and food intake. Mice in the NLD group were not included in statistical analysis of plaque composition. Statistical significance was considered if *p* < 0.05.

## Results

### Animal characteristics, body weight and food intake

**Fig. 1:**
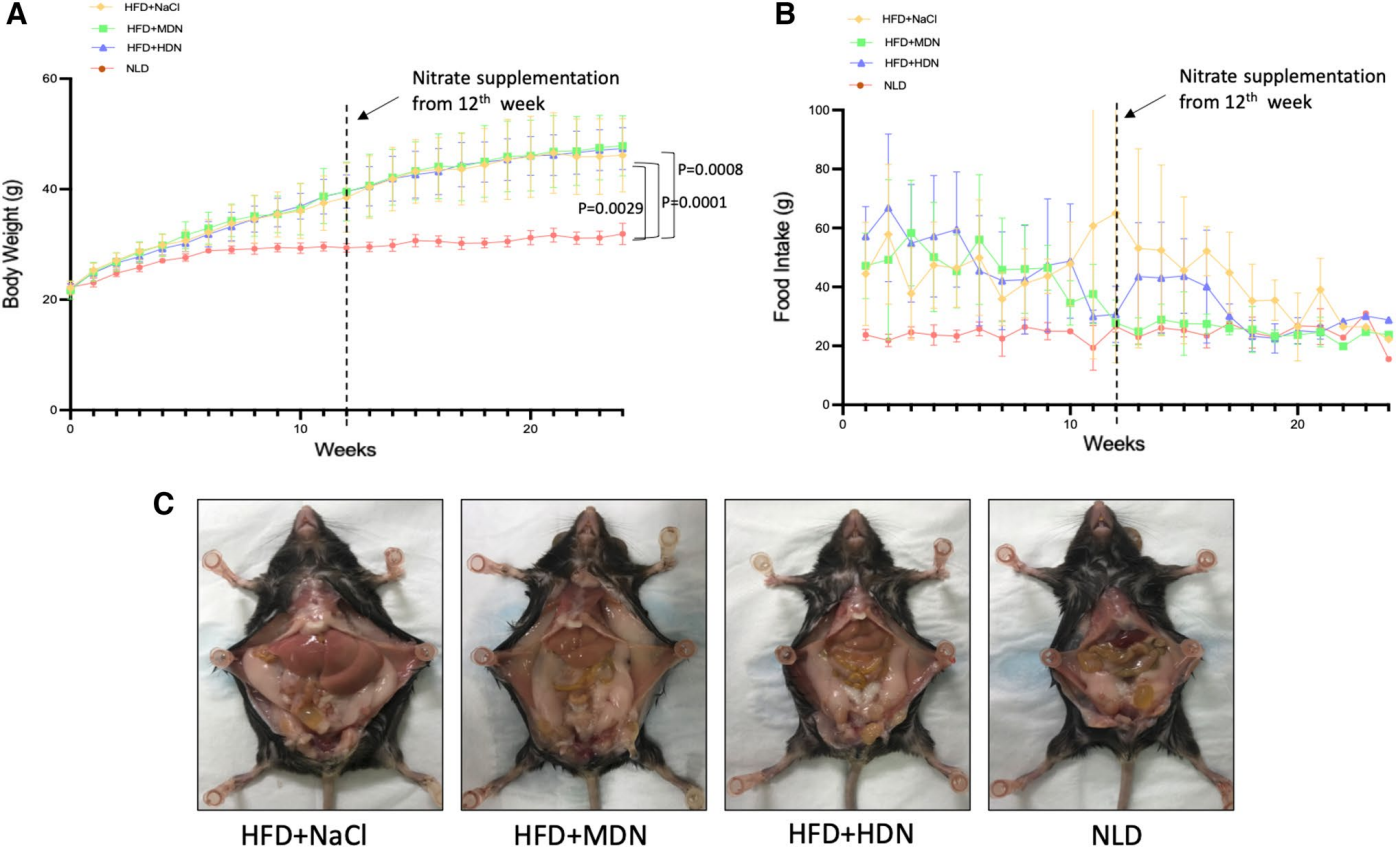
Effects of nitrate on **A** body weight, **B** food intake, and **C** visceral fat in apoE^−/−^ mice fed a normal chow diet or a high fat diet with or without dietary nitrate. Mean ± SEM (n = 15/group for body weight and food intake, n = 10/group for visceral fat). Repeated measures analysis, **A** p < 0.0001 for all groups vs NLD. ANOVA with post-hoc analysis for mean weight at 24 weeks, HFD+NaCl vs. NLD, p = 0.0029; HFD + MDN vs. NLD, p = 0.0008; HFD + HDN vs. NLD, p = 0.0001. **B** Not significantly different for repeated measures analysis. *HFD* high fat diet, *HDN* high dose nitrate, *MDN* moderate dose nitrate, *NLD* normal laboratory diet

Nine mice; one in the HFD+HDN group, four in the HFD+MDN group, and four in the HFD+NaCl group had to be sacrificed prior to the end of the study due to the development of severe dermatitis. Analysis of the water determined that the moderate-dose nitrate contained 130 μg/mL of nitrate and 3 μg/mL of nitrite, and the high-dose nitrate contained 1301 μg/mL of nitrate and 26.6 μg/mL of nitrite [18]. This corresponds to a 1 mmol/kg/day and 10 mmol/kg/day intake respectively, assuming mice drink 5–6 mL water/day [23]. As expected, apoE^−/−^ mice fed the HFD had a significant increase in body weight gain compared to mice consuming the NLD over 24 weeks and in mean body weight at the end of the study period (Fig. 1A). There was no significant difference in body weight gain or mean body weight at 24 weeks between apoE^−/−^ mice fed the HFD alone or those supplemented with moderate or high dose nitrate. Average food consumption (g/mouse/week) was not significantly different in mice consuming any of the diets (Fig. 1B).

### Circulating nitrate and nitrite

Supplementation with moderate or high dose nitrate significantly increased serum concentration of nitrate (n = 10/group, control group: 33.12 ± 3.87 μM; HFD + MDN group: 136.52 ± 30.24 μM; HFD + HDN group: 1020.24 ± 473.58 μM, *p* < 0.0001) and nitrite (control group: 2.95 ± 0.27 μM; HFD + MDN group: 7.33 ± 1.63 μM; HFD + HDN group: 36.71 ± 13.58 μM, *p* < 0.0001). The mice supplemented with high dose nitrate had significantly higher circulating concentrations of both nitrate and nitrite than the moderate dose group (nitrate: *p* < 0.0001; nitrite: *p* < 0.0001) (Supplementary Fig. 2).

### Visceral fat accumulation and serum lipid profile

ApoE^−/−^ mice fed a HFD had increased accumulation of visceral fat around the central torso region compared to mice fed a NLD. Fat accumulation and adipocyte size were not affected by nitrate supplementation (Fig. 1C and Supplemental Fig. 1 ). The HFD significantly increased serum levels of TG, TC and LDL-C compared to the NLD (Table 1). High dose nitrate supplementation significantly attenuated the HFD-induced increase in serum TG (2.00 ± 0.56 vs 2.53 ± 0.76 mmol/L), but there was no effect of nitrate at either dose on serum TC or LDL-C. There were no significant changes in HDL-C among the treatment groups. As previously reported, hepatic lipid accumulation was significantly increased in mice fed the HFD, which was reduced with both high and moderate dose nitrate [24].

**Table 1:** Serum lipid profile in apoE^−/−^ mice fed a high fat or normal laboratory diet at 24 weeks of intervention

	HFD + NaCl	HFD + MDN	HFD + HDN	NLD
TG (mmol/L)	2.53 ± 0.76*	2.07 ± 0.68*	2.00 ± 0.56*^#^	1.33 ± 0.71
TC (mmol/L)	31.82 ± 7.83*	35.87 ± 4.88*	41.01 ± 8.70*	13.94 ± 2.55
LDL-C (mmol/L)	10.18 ± 1.66*	10.55 ± 3.24^*^	10.68 ± 2.93*	2.18 ± 0.67
HDL-C (mmol/L)	0.68 ± 0.19	0.62 ± 0.17	0.56 ± 0.17	0.56 ± 0.17

Data are presented as mean ± SEM. N = 8 in all HFD groups and n = 10 in NLD group

*TC* total cholesterol, *TG* triglycerides, *HDL-C* high-density lipoprotein cholesterol, *HFD* high fat diet, *HDN* high dose nitrate, *LDL-C* low-density lipoprotein cholesterol, *MDN* moderate dose nitrate, *NLD* normal laboratory diet

*p < 0.05 compared with NLD

^#^*p* < 0.05 compared with HFD+NaCl

### Serum levels of ET-1, leptin and cGMP

**Fig. 2:**
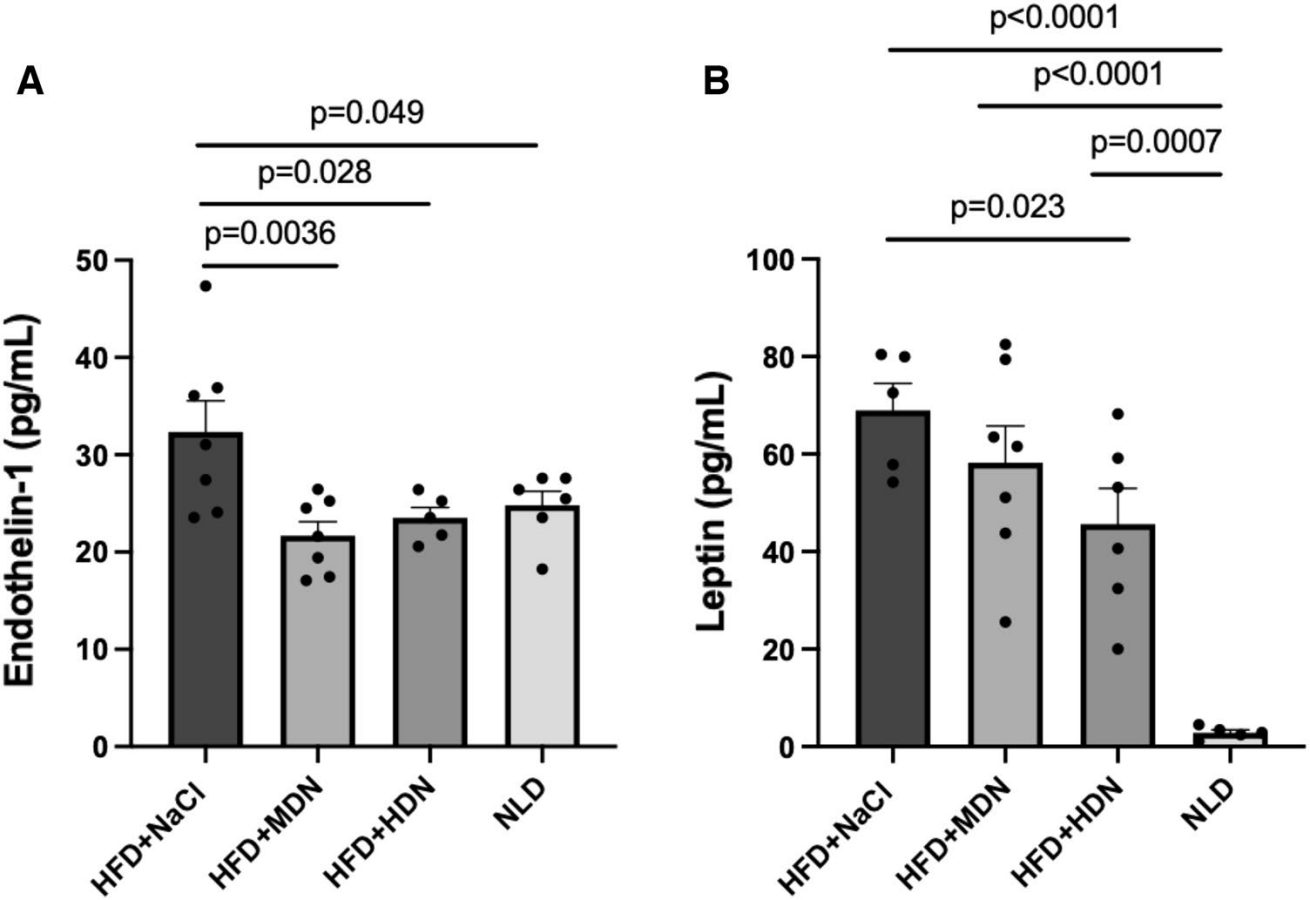
Effects of nitrate on serum **A** endothelin-1 and **B** leptin in apoE^−/−^ mice fed a normal laboratory diet or a high fat diet with or without dietary nitrate. Mean ± SEM (Endothelin-1: n = 7 in HFD+NaCl, n = 7 in HFD + MDN, n = 5 in HFD + HDN, n = 6 in NLD. Leptin: n = 5 in HFD+NaCl, n = 7 in HFD+MDN, n = 6 in HFD + HDN, n = 5 in NLD). Serum endothelin-1: p = 0.049 HFD+NaCl vs. NLD; p = 0.0028 HFD+NaCl vs. HFD+HDN; p = 0.0036 HFD+NaCl vs. HFD+MDN. Serum leptin: p < 0.0001 HFD+NaCl vs. NLD; p < 0.0001 HFD+MDN vs. NLD; p = 0.0007 HFD+HDN vs. NLD; p = 0.023 HFD+NaCl vs. HFD+HDN. *HFD* high fat diet, *HDN* high dose nitrate, *MDN* moderate dose nitrate, *NLD* normal laboratory diet

Serum ET-1 was significantly elevated in apoE^−/−^ mice fed a HFD compared with the NLD (Fig. 2A). Supplementation with both moderate and high dose nitrate significantly reduced serum ET-1 concentrations to levels comparable to the NLD (Fig. 2A). Serum leptin concentrations were significantly elevated with the HFD relative to the NLD (Fig. 2B). High dose nitrate supplementation significantly attenuated the increase in serum leptin concentration relative to the HFD control diet. There were no significant differences in serum cGMP concentration between the treatment groups (data not shown).

### Atherosclerotic lesion size and composition

**Fig. 3:**
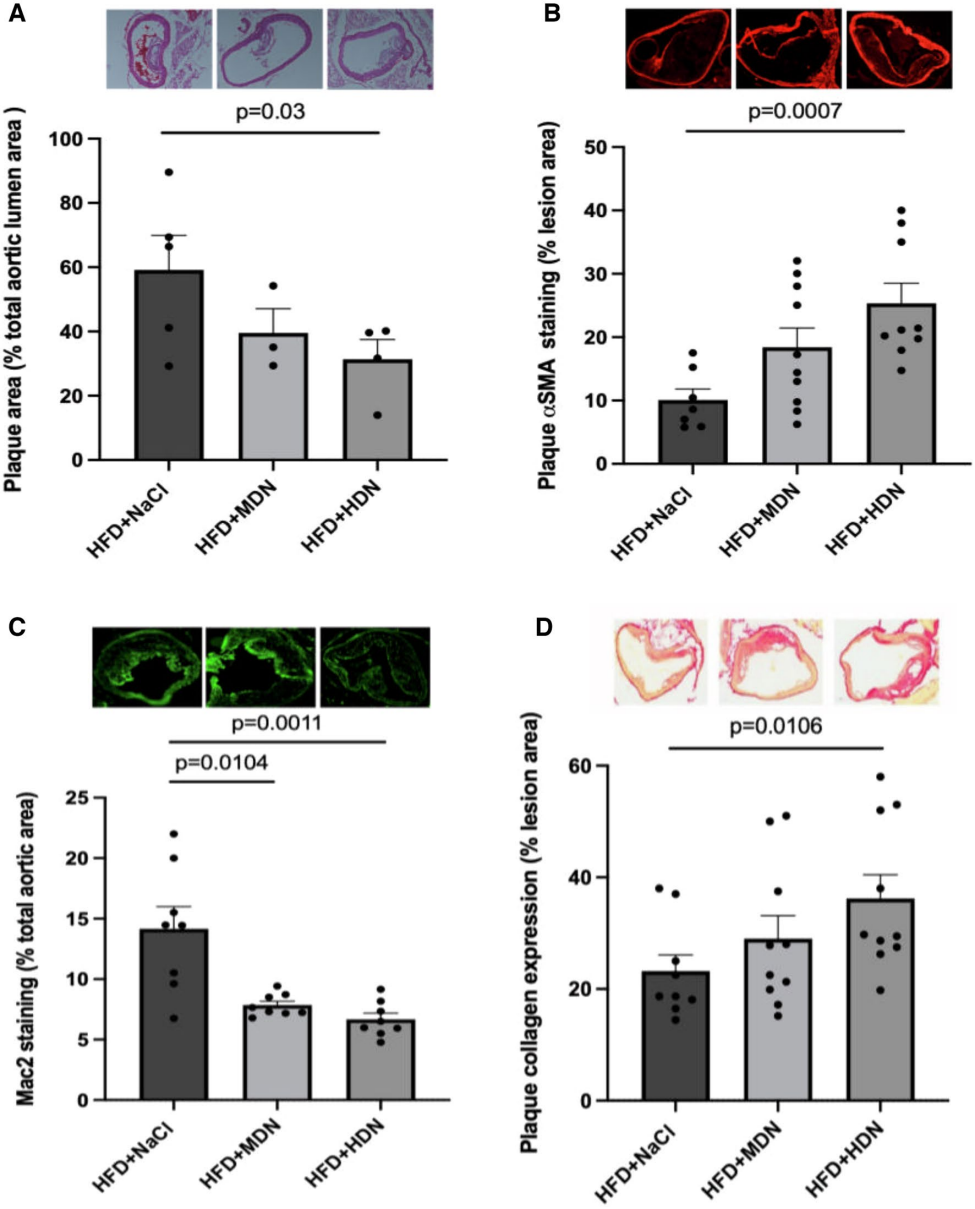

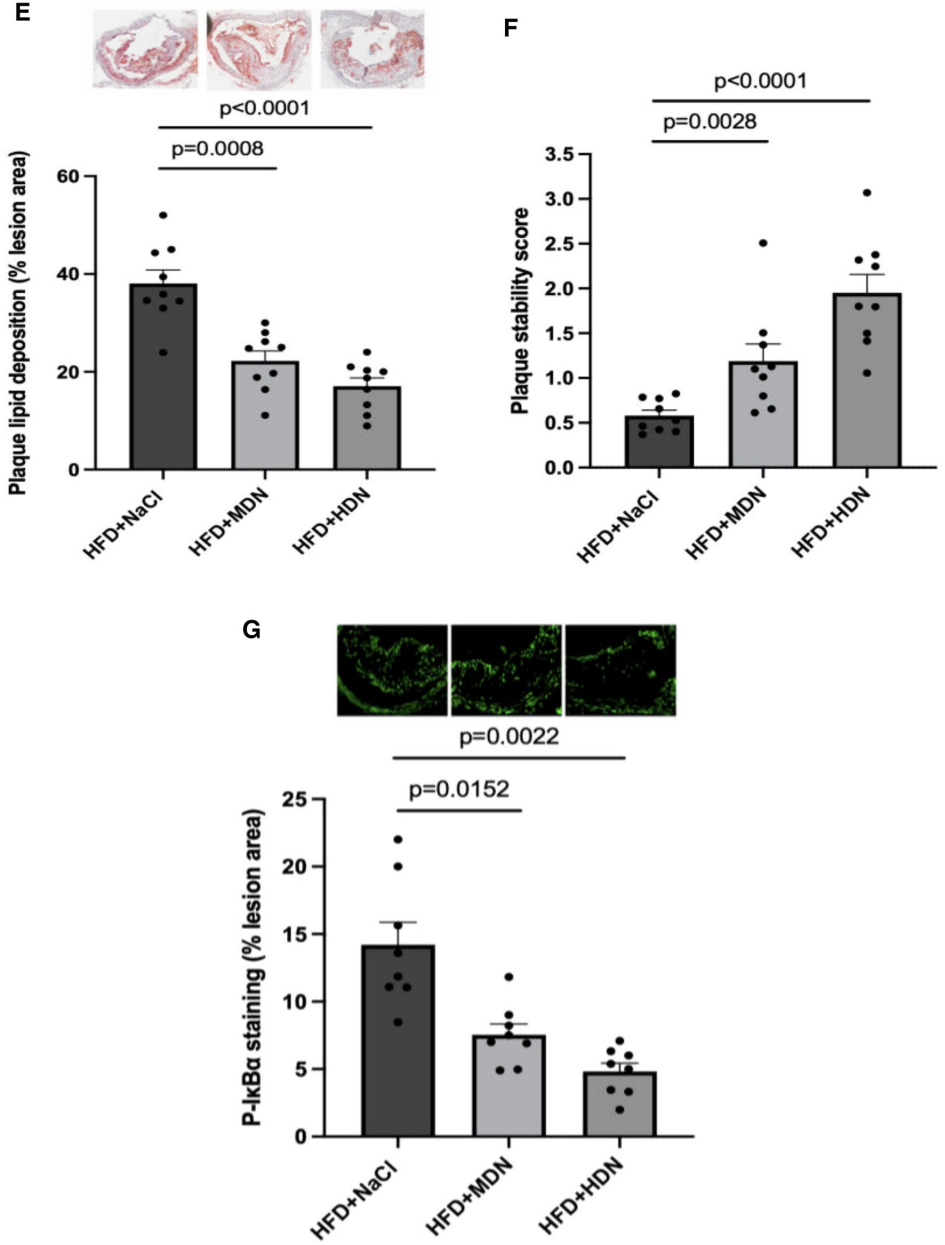
Representative images and quantification of **A** total plaque area, **B** αSMA staining, **C** CD63 staining, **D** picrosirius red staining, **E** Oil Red O staining, **F** plaque stability scor, and **G** pIκBα staining from apoE^−/−^ mice fed a normal laboratory diet or high fat diet with or without dietary nitrate. Mean ± SEM (Plaque area: n = 5 in HFD+NaCl, n = 3 in HFD+MDN, n = 4 in HFD+HDN. αSMA: n = 7 in HFD+NaCl, n = 10 in HFD+MDN, n = 9 in HFD+HDN. Mac2 staining: n = 8 for all groups. Collagen assessment: n = 9 in HFD+NaCl, n = 10 in HFD+MDN, n = 10 for HFD+HDN. Lipid deposition: n = 9 for all groups. Plaque stability: n = 9 for all groups. pI_K_Bα: n = 8 for all groups). Plaque area: p = 0.03 HFD+NaCl vs. HFD+HDN; Plaque αSMA: p = 0.0007 HFD+NaCl vs. HFD+HDN; Mac2: p = 0.0104 HFD+NaCl vs. HFD+MDN, p = 0.0011 HFD+NaCl vs. HFD+HDN; Plaque collagen: p = 0.0106 HFD+NaCl vs. HFD+HDN; Plaque lipid: p = 0.0008 HFD+NaCl vs. HFD+MDN, p < 0.0001 HFD+NaCl vs. HFD+HDN; Plaque stability score: p = 0.0028 HFD+NaCl vs. HFD+MDN, p < 0.0001 HFD+NaCl vs. HFD+HDN; pIκBα: p = 0.0152 HFD+NaCl vs. HFD+MDN, p = 0.0022 HFD + NaCl vs. HFD+HDN. *HFD* high fat diet, *HDN* high dose nitrate, *MDN* moderate dose nitrate. Mice fed a NLD were not included in this analysis as no plaque was detected

Atherosclerotic lesions were significantly developed following 24 weeks of HFD. Supplementation with high dose nitrate significantly reduced lesion area, by approximately 50%, compared to the HFD+NaCl control group, with non-significant reductions observed in the moderate dose nitrate group (Fig. 3A). To evaluate the infiltration of vascular smooth muscle cells (VSMCs) in the atherosclerotic lesions, immunostaining for αSMA was performed. αSMA-positive regions in the mice supplemented with high dose nitrate were significantly greater compared to those in the control group (Fig. 3B). The degree of infiltrated macrophages was assessed by CD68 staining. The lesion areas positive for CD68 was significantly reduced in both moderate and high dose nitrate groups compared with HFD+NaCl controls (Fig. 3C). Moreover, high dose nitrate increased plaque collagen expression (Fig. 3D), and both moderate and high dose nitrate supplementation reduced plaque lipid deposition (Fig. 3E). Accordingly, the histological plaque stability score, calculated as the collagen:lipid ratio, was significantly greater in the moderate and high dose nitrate treated mice (Fig. 3F). P-IκBα staining, indicative of NFκB activation and inflammation, was significantly lower in the lesions of the mice supplemented with moderate and high dose nitrate compared to the HFD+NaCl control mice (Fig. 3G).

### Tissue protein expression

**Fig. 4:**
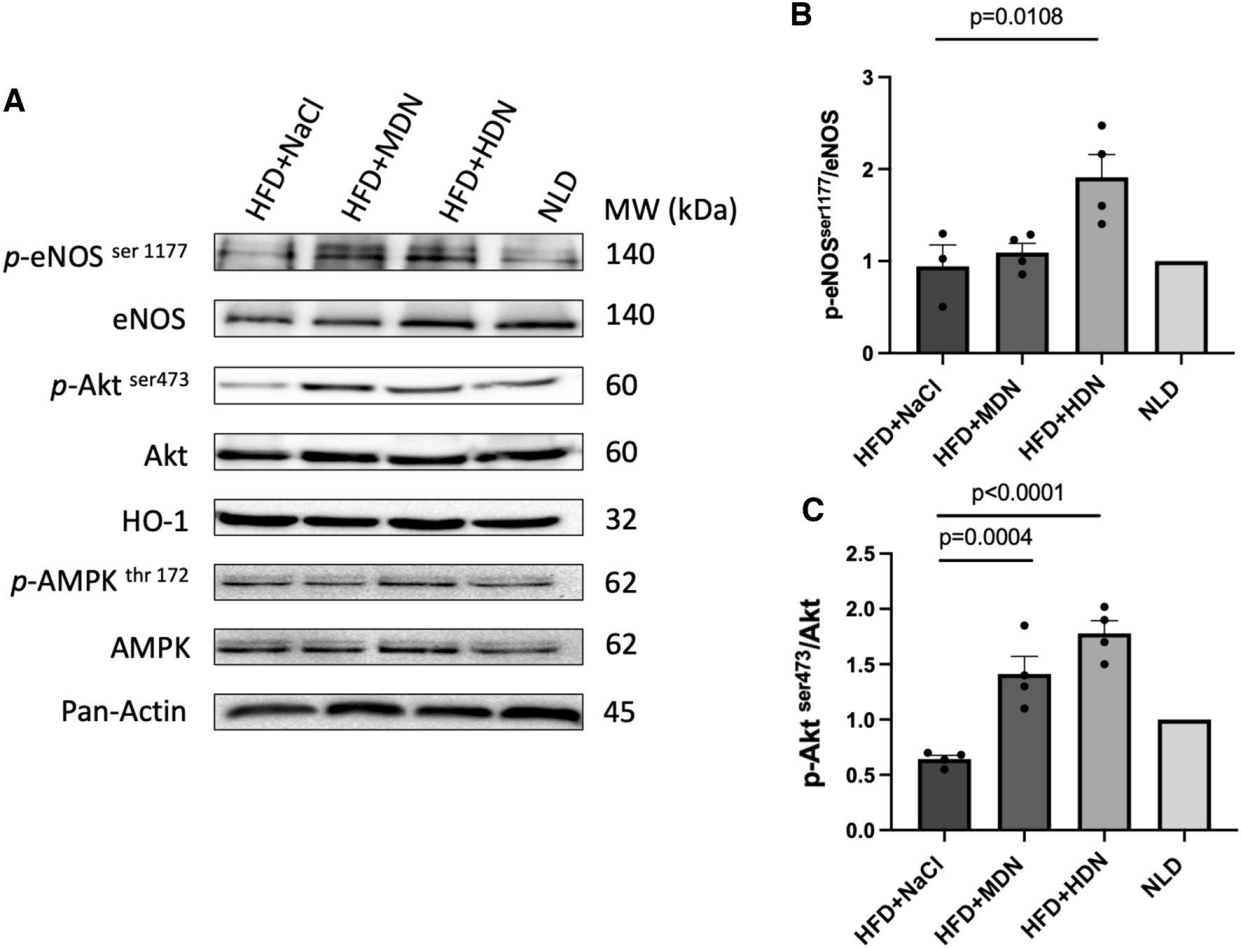
**A** Aortic expression and **B**, **C** quantification of eNOS, p-eNOS^ser1177^, Akt, p-Akt^ser47^, HO-1, AMPK, p-AMPK^thr172^, and pan-actin expression in apoE^−/−^ mice fed a normal laboratory diet or high fat diet with or without dietary nitrate. Mean ± SEM (p-eNOS/eNOS: n = 3 in HFD+NaCl, n = 4 in HFD+MDN, n = 4 in HFD+HDN, n = 3 in NLD. P-Akt/Akt: n = 4 in HFD+NaCl, n = 4 in HFD+MDN, n = 4 in HFD+HDN, n = 3 in NLD). p-eNOS^ser1177^/ eNOS: p = 0.0108 HFD+NaCl vs. HFD+HDN; p-Akt^ser47^/ Akt: p < 0.0001 HFD + NaCl vs. HFD+HDN; p = 0.0004 HFD+NaCl vs. HFD+MDN. *HFD* high fat diet, *HDN* high dose nitrate, *MDN* moderate dose nitrate, *NLD* normal laboratory diet

**Fig. 5:**
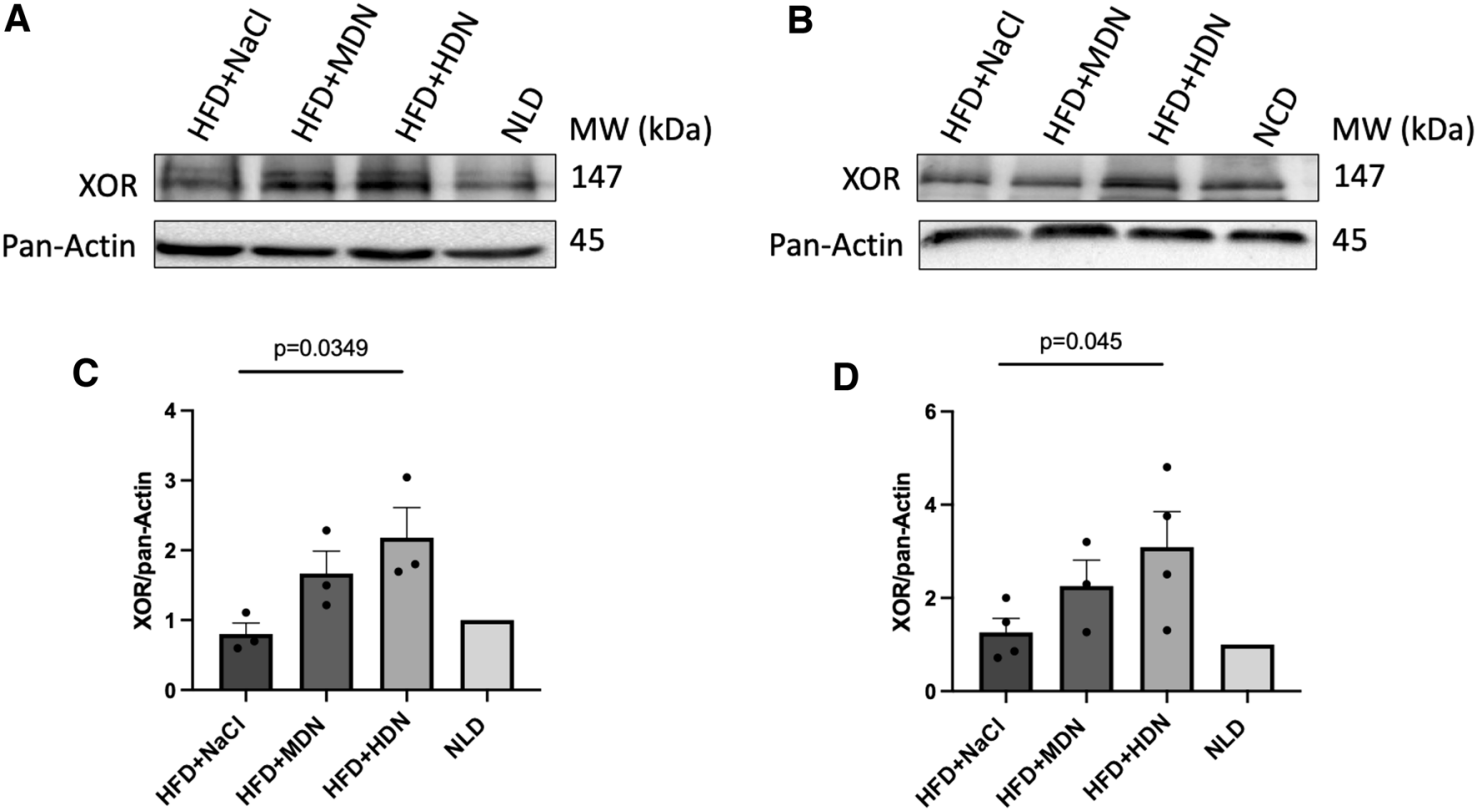
XOR expression in (**A**, **C**) aorta and **B**, **D** liver of apoE^−/−^ mice fed a normal laboratory diet or high fat diet with or without dietary nitrate. Mean ± SEM (Aorta: n = 3 for all groups. Liver: n = 4 in HFD + NaCl, n = 3 in HFD + MDN, n = 4 in HFD + HDN, n = 3 in NLD). XOR/pan-Actin (aorta): p = 0.0349 HFD+NaCl vs. HFD+HDN; XOR/pan-Actin (liver): p = 0.045 HFD+NaCl vs. HFD+HDN. *HFD* high fat diet, *HDN* high dose nitrate, *MDN* moderate dose nitrate, *NLD* normal laboratory diet

No significant difference in the aortic expression of total eNOS, AMPK, Akt, and HO-1 in mice fed the HFD supplemented with either dose of nitrate was observed (Fig. 4A). We observed a significant increase in the *p*-eNOS ^ser1177^/eNOS ratio in high dose nitrate-treated groups compared to the HFD control group (Fig. 4A, B). There was no effect on the aortic p-AMPK^thr172^/AMPK ratio (Fig. 4A). A significant dose-dependent increase in p-Akt^ser473^/Akt ratio was observed in mice receiving the nitrate compared to the HFD control group (Fig. 4A, C). XOR expression in both the aorta and liver of mice supplemented with high dose nitrate was significantly increased compared with the HFD control group (Fig. 5).

## Discussion

In the present study we have demonstrated a significant attenuation in the progression of established atherosclerosis in apoE^−/−^ mice fed a HFD supplemented with nitrate. These beneficial effects appeared more pronounced in the high dose nitrate group. This improvement was associated with increased collagen expression and decreased macrophage and lipid deposition, suggesting an increase in overall plaque stability. In addition, there were significant reductions in plaque size and circulating endothelin-1 and triglycerides following high dose nitrate supplementation. Although the high dose nitrate would be difficult to achieve through dietary changes alone, it may be possible through the use of supplements and thus represents a potential new treatment option. To our knowledge, this is the first study demonstrating a beneficial effect of chronic high dose nitrate supplementation on established atherosclerosis in the apoE^−/−^ mouse model.

Our previous study demonstrated a beneficial effect of low (0.1 mmol/kg/day) and moderate (1 mmol/kg/day) dose nitrate in preventing the development of atherosclerosis in the apoE^−/−^ mouse, with no additional benefit observed with the high dose nitrate [18]. In the present study, a moderate and high dose of nitrate were investigated to determine any beneficial effect after atherosclerosis is already established. Despite the lack of a beneficial effect of the high dose nitrate in our previous study [18], the high dose was included in the present study in order to determine if higher doses were required when investigating a model of established disease. Indeed, our data show that the greatest effects on plaque size and composition were observed after supplementation with high dose nitrate, suggesting that preventing or reversing the progression of established atherosclerosis may require significantly greater doses of nitrate.

As expected, the HFD progressively increased body weight compared to the normal chow diet, due to the accumulation of fat mass. Nitrate treatment, at either dose, had no effect on body weight or fat accumulation. Previous studies have shown that dietary supplementation with inorganic nitrate is associated with decreased body weight [25, 26], although in these studies, mice were supplemented with nitrate from the beginning of the HFD treatment. In this current study, nitrate was supplemented in the diet at week 12 when the mice already had significant weight gain. Previous studies have demonstrated no effect of nitrate on weight in mouse models of atherosclerosis or the metabolic syndrome [18, 27]. Significant increases were also observed in TC, LDL and TG in mice fed the HFD, confirming this animal as a model of atherosclerosis. Despite this, only TG levels were significantly decreased in mice supplemented with high dose nitrate. Consistent with our finding, decreased serum TG in eNOS-deficient mice after dietary nitrate administration have previously been reported by Carlstrom’s group [26]. Elevated TG levels are observed in atherosclerosis and are recognized as a treatment target to lower cardiovascular risk [28]. Therefore, the reduction of TG levels with nitrate treatment may indicate a novel pathway by which nitrate/nitrite may affect fat metabolism or utilization of energy. Further work to investigate the beneficial effects of chronic nitrate supplementation on TC and LDL may be required.

Endothelial dysfunction is recognised as an initial first step in the development of atherosclerosis and is characterised by reduced vascular flow responses as well as lowered circulating NO levels [29]. In this study, increased levels of serum nitrate and nitrite were observed in mice supplemented with nitrate, suggesting uptake and conversion of the nitrate from the drinking water. ET-1 is a peptide predominately produced in endothelial cells where it acts as a vasoconstrictor, pro-inflammatory factor, and platelet activator [30, 31]. As such, ET-1 and NO are natural counterparts in regard to vascular function, and an imbalance in the production of these two agents may contribute to the onset of vascular dysfunction and subsequent atherosclerosis. In the present study, the HFD contributed to a significant increase in serum ET-1 levels in apoE^−/−^ mice, indicative of endothelial dysfunction. Supplementation with nitrate, at both moderate and high doses, significantly reduced serum ET-1 levels, suggesting a protective effect on vascular function, possibly via conversion of the nitrate to nitrite and NO.

At the early stage of atherogenesis, the adherence of inflammatory cells enriched in lipids to the damaged endothelium results in the formation of a lipid-rich core. If inflammatory conditions persist, the lipid core continues to grow. Subsequently, activated leukocytes secrete proteases to degrade the extracellular matrix, meanwhile pro-inflammatory cytokines limit the synthesis of new collagen. These changes induce a thin fibrous cap and increase the risk of plaque rupture [32]. As expected, the HFD increased plaque burden compared to mice fed the normal chow diet. Supplementation with high dose nitrate reduced the total plaque burden in the HFD fed apoE^−/−^ mice. Further investigation of plaque composition revealed that nitrate supplementation was associated with a reduction in macrophage accumulation and lipid deposition within the plaque. This change was associated with increased SMC accumulation and collagen expression within the plaque, suggestive of an increase in plaque stability [33]. The effect of nitrate on smooth muscle cell accumulation may seem counterintuitive, as it has long been proposed that NO exerts inhibitory effects on VSMC proliferation, and that nitrite also inhibits smooth muscle proliferation in models of vascular injuries [34–36]. However, we speculate that the accumulation of smooth muscle within the plaque is likely to be caused by the reduced macrophage content via an indirect pathway. Indeed, it has been previously demonstrated that SMC proliferation is inhibited when co-cultured with macrophages [37], suggesting that reducing the macrophage accumulation in the plaque results in a consequent removal of the inhibitory influence on SMC proliferation. The clinical implications of this finding however, remain to be elucidated.

Leptin has previously been implicated in the development of atherosclerosis due to the presence of the leptin receptor in atherosclerotic lesions [38]. High levels of leptin have been shown to increase oxidative stress in endothelial cells [39], favour VSMC migration and proliferation [40], decrease arterial distensibility [41], and contribute to obesity-associated hypertension [42]. All these effects have been found to be inversely associated with vascular health and strongly related to the pathophysiology of atherosclerosis. A previous animal model observed lower plasma leptin levels in the nitrate-fed group compared to controls [43]. Supporting this finding, we observed significantly increased serum leptin in apoE^−/−^ mice fed a HFD, which was attenuated following high dose nitrate supplementation.

XOR has been proposed to be a major source of reactive oxygen species (ROS) and emerging evidence has suggested that XOR mediates NO formation by reducing inorganic nitrate and nitrite [44]. Several reports demonstrate a significant elevation in XOR activity and expression in models of atherosclerosis [45] as well as within plaques isolated from human patients [46]. While XOR is highly activated in liver and intestine, human endothelial cells from the microvasculature of several tissues also have high levels of XOR activity [47]. The hypoxic environment within the plaque represents an ideal environment for nitrite reduction and in particular, provides a condition to potentiate XOR-dependent nitrite reduction. The present study showed elevated XOR expression in both the liver and aorta of mice supplemented with high dose nitrate, thereby suggesting that in atherosclerosis, nitrite/nitrate bioactivity is enhanced due to the up-regulated XOR-dependent nitrite reductase activity. Previous studies in ischaemia–reperfusion injury, have shown nitrate supplementation increased nitrite reductase activity by XOR produces NO, which may protect against further injury [48]. This protection has been attributed to the inhibition of the mitochondrial respiration that limited ROS production and improved myocardial vascularization [5]. The increased expression of XOR in the present study suggests it may play a role in the protective effects of nitrate, however further work inhibiting the XOR pathway is required.

Within the body, eNOS activity is induced by various chemical factors or mechanical stimuli, which then stimulate kinases to phosphorylate eNOS, leading to an increase or decrease in eNOS activity [49]. Previous studies have demonstrated that a variety of atherogenic stimuli suppress eNOS protein levels in cultured endothelial cells [50, 51]. A significant decrease was identified in eNOS gene expression in human aortic and coronary endothelial cells from advanced atherosclerotic lesions, but not in those of early atherosclerotic samples [52]. Consistent with our findings, invesitigations in atherosclerotic animal models demonstrated unchanged or even augmented expression of eNOS in atherosclerotic arteries, despite the presence of endothelial dysfunction [53, 54]. One recent study in human coronary atherectomy specimens revealed a higher eNOS gene expression in patients with acute coronary syndromes compared to those with stable angina [55]. These results indicate that at least in the early stage of atherosclerosis, endothelial dysfunction is not attributable to a decreased expression of eNOS. Supporting this observation, we did not find significant differences in eNOS protein expression between the treatment groups. Protein phosphorylation, a key regulator of eNOS activity, is modulated by kinases, phosphatases and protein–protein interactions. The serine/threonine kinase Akt (protein kinase B), a multifunctional serine/threonine kinase, can directly phosphorylate eNOS at the serine 1177 residue, activate the enzyme and faciliate NO production [56]. In the present study, aortic eNOS and Akt activity, as demonstrated by increased phopshorlyation of these proteins, was increased with high dose nitrate supplementation. These results suggest that long term nitrate supplementation may stimulate vascular endothelial cells to produce NO via the upregulation of eNOS activity. However, it should be noted that further studies need to be conducted to ascertain the translation of our findings to humans, as it’s highly likely that species differ in both their response to and metabolism of nitrate. While the dose used in the present study is high and unlikely to be achieved through dietary changes alone, the benefits when translating these findings to humans will also need to consider the currently recommended daily intakes of nitrate of International Food Commissions. Furthermore, while this present study was designed as a proof-of-principle study, using an animal model of atherosclerosis, future studies will need to demonstrate not only if these benefits translate but also determine a dose that is efficacious and safe. It is important to be mindful that any cardiovascular benefits need to be weighed up against the potential adverse carcinogenic effects of high-dose nitrate.

The present study has demonstrated that chronic high dose nitrate supplementation can attenuate the progression of established atherosclerosis in apoE^−/−^ mice. Mechanistically, this appears to be mediated through a XOR-dependent reduction of nitrite to NO, as well as enhanced eNOS activation via increased Akt and eNOS phosphorylation. Importantly, these beneficial effects of nitrate have been observed after disease has already been established, which has important implications when translating our findings to humans. Dietary and supplemental approaches to increase nitrate intake, which may have effects on both the nitrate-nitrite-NO pathway and eNOS-NO pathway may have therapeutic potential to attenuate atherosclerosis. While the present study suggests a potential cardioprotective effect from long-term nitrate supplementation, further work to investigate the translational aspects of these findings, including the appropriate dose as well as any potential detrimental effects of a nitrate dose outside current recommendations, in humans is required.

## Supplementary Information

Below is the link to the electronic supplementary material.

Supplementary file1 (DOCX 8179 KB)

## Data Availability

The datasets used and analysed in this current study are available from the corresponding author upon reasonable request.
