# Supplementary material for: Long-term dietary nitrate supplementation slows the progression of established atherosclerosis in ApoE−/− mice fed a high fat diet
Source: Eur J Nutr. 2023 Feb 28;62(4):1845–57. doi: 10.1007/s00394-023-03127-7 (PMC10195750; doi:10.1007/s00394-023-03127-7)
Supplement: Supplementary file 1 — Supplementary file1 (DOCX 8179 KB) [file 394_2023_3127_MOESM1_ESM.docx]

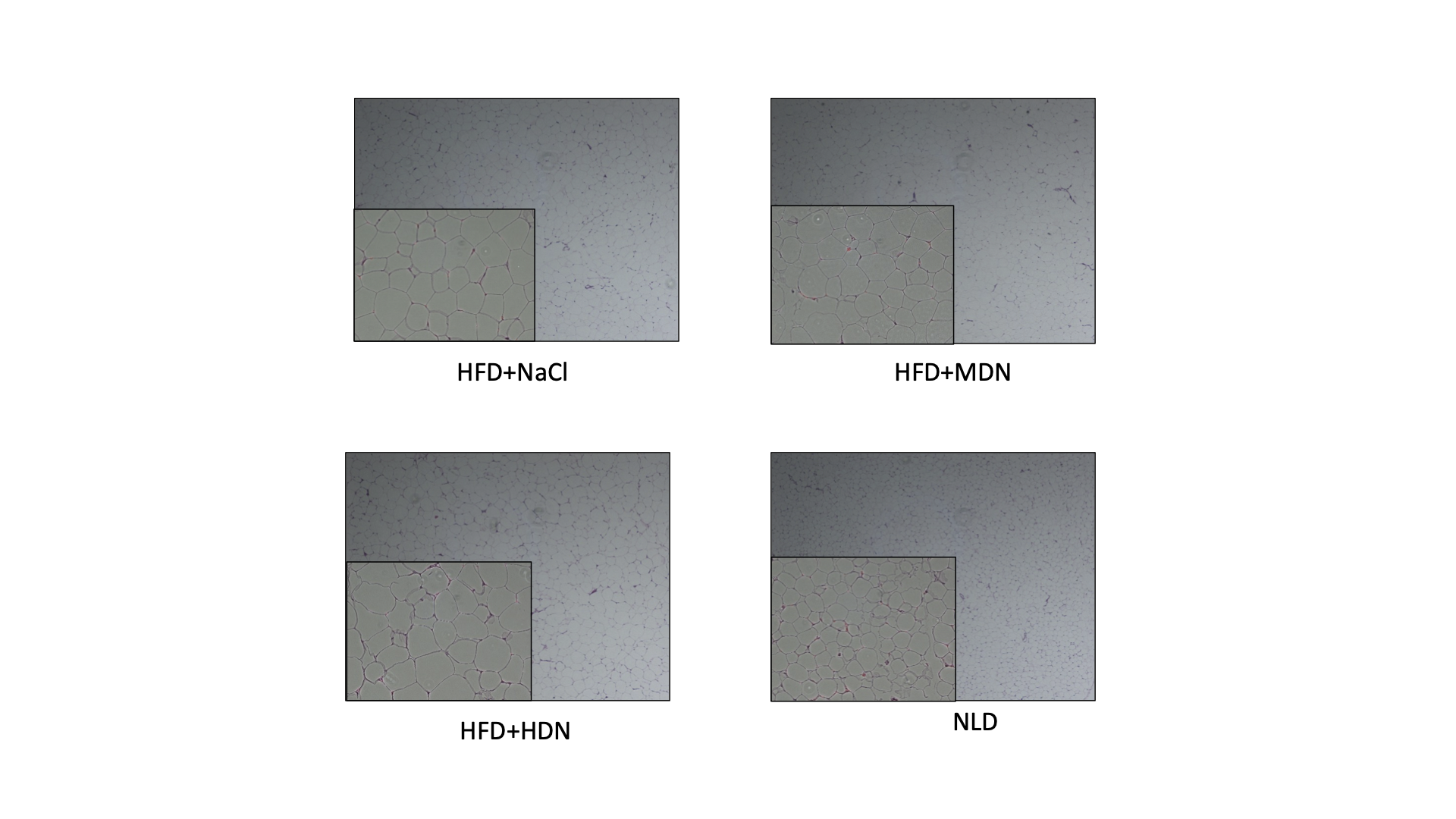


***Supplementary Figure 1:*** *Effects of nitrate on adipocyte hypertrophy in apoE-/- mice fed a normal chow diet or a high fat diet with or without dietary nitrate. Mean ± SEM (n=10/group). Not significantly differences in adipocyte size between the treatment groups. HFD, high fat diet; HDN, high dose nitrate; MDN, moderate dose nitrate; NLD, normal laboratory diet.*

***Figure 2****:* *GCMS analysis of serum nitrate (A) and nitrite (B) in HFD-fed ApoE^−/−^ mice. Results are mean ± SEM (n = 10 per group). ^#^p < 0.05 versus HFD+NaCl group. HFD, high fat diet; HDN, high dose nitrate; MDN, moderate dose nitrate; NLD, normal laboratory diet.*
